# La variété β-NaMoO_2_(AsO_4_)

**DOI:** 10.1107/S1600536809002517

**Published:** 2009-01-28

**Authors:** Soumaya Ben Hlila, Mohamed Faouzi Zid, Ahmed Driss

**Affiliations:** a Laboratoire de Matériaux et Cristallochimie, Faculté des Sciences, Université de Tunis–El Manar, 2092 El Manar, Tunis, Tunisie

## Abstract

The title compound, sodium dioxidomolybdenum(VI) arsenate(V), β-NaMoO_2_AsO_4_, was prepared by solid-state reaction at 953 K. In the crystal structure, the AsO_4_ tetra­hedra and MoO_6_ octa­hedra (both with *m* symmetry) share corner atoms to form a three-dimensional framework that delimits cavities parallel to [010] where disordered six-coordinated sodium cations (half-occupation) are located. Structural relationships between the different orthoarsenates of the *A*MoO_2_AsO_4_ series (*A* = Ag, Li, Na, K and Rb) are discussed.

## Littérature associée

Pour le contexte général du travail, voir: Brown & Altermatt (1985 ▶); Benhamada *et al.* (1992 ▶); Haddad *et al.* (1988 ▶); Harrison *et al.* (1994 ▶); Ledain *et al.* (1997 ▶); Piffard *et al.* (1985 ▶); Zid *et al.* (1992 ▶). La structure est isotypique avec AgMoO_2_AsO_4_ (Hajji & Zid, 2006 ▶) et KMoO_2_AsO_4_ (Zid & Jouini, 1996*a* ▶). Pour structures associées, voir: Hajji *et al.* (2004 ▶) (β-LiMoO_2_AsO_4_); Hajji *et al.* (2005 ▶) [Li(MoO_2_)_2_O(AsO_4_)]; Linnros (1970 ▶) (LiMoO_2_AsO_4_); Zid & Jouini (1996*b* ▶) (K_2_MoO_2_As_2_O_7_); Zid & Jouini (1999 ▶) (RbMoO_2_AsO_4_); Zid *et al.* (1997 ▶) (α-NaMoO_2_AsO_4_); Zid *et al.* (1998 ▶) (K_2_MoO_2_As_2_O_7_).

## Partie expérimentale

### 

#### Données cristallines

NaMoO_2_(AsO_4_)
*M* *_r_* = 289.85
Orthorhombique, 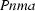

*a* = 10.147 (2) Å
*b* = 6.597 (2) Å
*c* = 7.420 (2) Å
*V* = 496.7 (2) Å^3^
*Z* = 4
Radiation Mo *K*α
μ = 9.29 mm^−1^
*T* = 298 (2) K
0.26 × 0.16 × 0.10 mm

#### Collection des données

Diffractomètre Enraf–Nonius CAD-4
Correction d’absorption: ψ scan (North *et al.*, 1968 ▶) *T*_min_ = 0.186, *T*_max_ = 0.394
1438 réflexions mesurées
589 réflexions independantes
539 réflexions avec *I* > 2σ(*I*)
*R*_int_ = 0.027
2 réflexions de référence fréquence: 120 min déclin d’intensité: 1.2%

#### Affinement

*R*[*F* ^2^ > 2σ(*F* ^2^)] = 0.019
*wR*(*F* ^2^) = 0.053
*S* = 1.14
589 réflexions
56 paramètres
Δρ_max_ = 0.80 e Å^−3^
Δρ_min_ = −0.69 e Å^−3^

### 

Collection des données: *CAD-4 EXPRESS* (Duisenberg, 1992 ▶; Macíček & Yordanov, 1992 ▶); affinement des paramètres de la maille: *CAD-4 EXPRESS*; réduction des données: *XCAD4* (Harms & Wocadlo, 1995 ▶); programme(s) pour la solution de la structure: *SHELXS97* (Sheldrick, 2008 ▶); programme(s) pour l’affinement de la structure: *SHELXL97* (Sheldrick, 2008 ▶); graphisme moléculaire: *DIAMOND* (Brandenburg, 1998 ▶); logiciel utilisé pour préparer le matériel pour publication: *WinGX* (Farrugia, 1999 ▶).

## Supplementary Material

Crystal structure: contains datablocks I, global. DOI: 10.1107/S1600536809002517/wm2218sup1.cif

Structure factors: contains datablocks I. DOI: 10.1107/S1600536809002517/wm2218Isup2.hkl

Additional supplementary materials:  crystallographic information; 3D view; checkCIF report

## Figures and Tables

**Table 1:** Paramètres géométriques(Å)

Mo1—O4	1.700 (3)
Mo1—O1^i^	1.714 (3)
Mo1—O5^ii^	1.999 (3)
Mo1—O5^iii^	1.999 (3)
Mo1—O3	2.174 (3)
Mo1—O2	2.197 (3)
As1—O2^i^	1.678 (3)
As1—O3^iv^	1.678 (3)
As1—O5	1.700 (2)
As1—O5^v^	1.700 (2)
Na1—O1	2.329 (4)
Na1—O2^vi^	2.420 (4)
Na1—O5^vii^	2.583 (4)
Na1—O4	2.590 (4)
Na1—O1^viii^	2.595 (4)
Na1—O3^vi^	2.597 (4)

Codes de symétrie: (i) 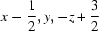; (ii) 
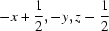
; (iii) 
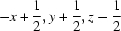
; (iv) 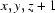; (v) 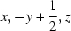; (vi) 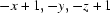; (vii) 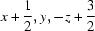; (viii) 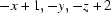.

**Fig. 1.**
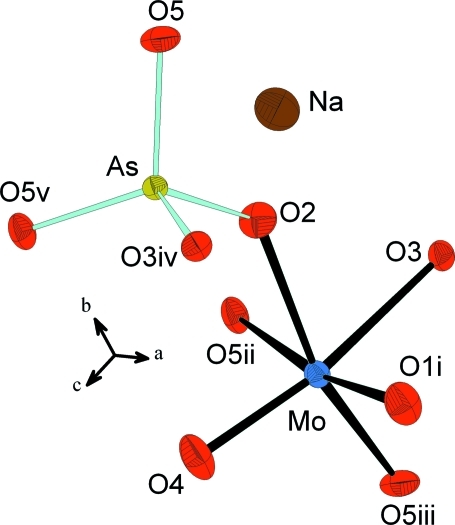
Unité asymétrique dans la variété β-NaMoO2AsO4. [Code de sym'etrie: voir table 1.]

**Fig. 2.**
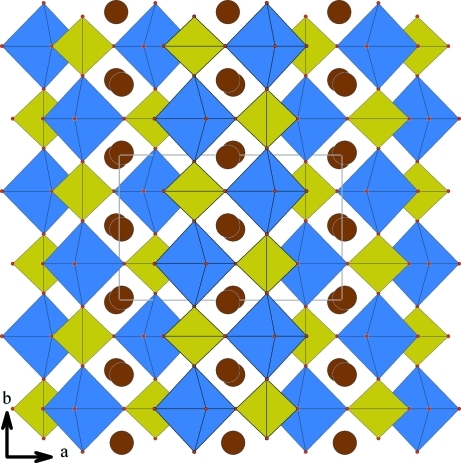
Projection de la structure de la variété β-NaMoO2AsO4 selon c montrant les châines MoAsO8.

**Fig. 3.**
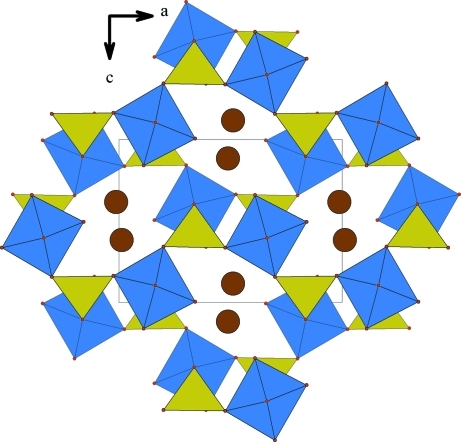
Projection de la structure de la variété β-NaMoO2AsO4 selon b montrant les canaux où résident les cations Na+.

## supplementary crystallographic information

### Comment

La recherche de nouveaux matériaux pouvant être potentiellement des conducteurs ioniques ou bien des échangeurs d'ions, a conduit à s'intéresser aux composés à charpentes mixtes formées d'octaèdres *M*O_6_ (*M* = métal de transition) et de tétraèdres *X*O_4_ (*X* = P, As). En effet, la jonction entre ces polyèdres conduit à des composés à charpentes ouvertes mixtes présentant de nombreuses propriétés physico-chimiques intéressantes qui sont en relation directe avec leurs structures cristallines (Benhamada *et al.*, 1992; Harrison *et al.*, 1994; Zid *et al.*, 1992; Piffard *et al.*, 1985; Haddad *et al.*, 1988; Ledain *et al.*, 1997). C'est dans ce cadre que nous avons exploré les systèmes *A*–Mo–As–O (*A* = cation monovalent) dans lesquels nous avons précédemment caractérisé les phases suivantes: Rb_2_MoO_2_As_2_O_7_ (Zid *et al.*, 1998), K_2_MoO_2_As_2_O_7_ (Zid & Jouini, 1996*b*), Li(MoO_2_)_2_O(AsO_4_) (Hajji *et al.*, 2005) et AgMoO_2_AsO_4_ (Hajji & Zid, 2006). La variété β-NaMoO_2_AsO_4_ obtenue appartient à la série des orthoarséniates de formulation générale *A*MoO_2_AsO_4_.

L'unité asymétrique (Fig. 1) est construite à partir d'un octaèdre MoO_6_ est d'un tétraèdre AsO_4_ partageant un sommet. Ces polyèdres se lient, selon les directions [100] et [010] pour former des chaînes ondulées de formulation MoAsO_8_ (Fig. 2). Ces dernières se connectent par mise en commun de sommets entre octaèdres et tétraèdres pour conduire à une charpente tridimensionnelle possédant des larges canaux où résident les cations Na^+^ (Fig. 3). Le calcul des différentes valences des liaisons utilisant la formule empirique (Brown & Altermatt, 1985) vérifie bien les valeurs de charges des ions Mo (5,94), As (4,95) et Na (0,90) dans la phase étudiée. La structure est similaire à celle au potassium KMoO_2_AsO_4_ (Zid & Jouini, 1996a) et à l'argent AgMoO_2_AsO_4_ (Hajji & Zid, 2006). Notons que dans la série *A*MoO_2_AsO_4_ le cation monovalent joue un rôle important et conduit à différents types structuraux notamment: LiMoO_2_AsO_4_ orthorhombique, groupe non-centrosymétrique *Pn*2_1_*a* (Linnros, 1970), NaMoO_2_AsO_4_ orthorhombique, groupe non-centrosymétrique *Pca*2_1_ (Zid *et al.*, 1997), RbMoO_2_AsO_4_ orthorhombique, groupe *Fddd* (Zid & Jouini, 1999), β-LiMoO_2_AsO_4_ monoclinique, groupe non-centrosymétrique *P*2_1_ (Hajji *et al.*, 2004) et la nouvelle variété β-NaMoO_2_AsO_4_ orthorhombique, groupe *Pnma*. Afin d'utiliser les données structurales et les relier aux propriétés physico-chimiques, en particulier de conduction ioniques et dès l'obtention d'une phase polycristalline pure du β-NaMoO_2_AsO_4_, des mesures éléctriques moyennant un pont d'impédance complexe de type HP4192A seront réalisées.

### Experimental

Les cristaux relatifs à la variété β-NaMoO_2_AsO_4_ ont été obtenus à partir des réactifs: (NH_4_)_2_Mo_4_O_13_ (Fluka), NH_4_H_2_AsO_4_ (préparé au laboratoire, ASTM 01-775) et Na_2_CO_3_ (Prolabo) pris dans les rapports molaires Na:Mo:As égaux à 2:3:4. Le mélange, finement broyé, est préchauffé à l'air à 623 K en vue d'éliminer NH_3_, H_2_O et CO_2_. Il est ensuite porté jusqu'à une température de synthèse proche de la fusion, 953 K. Le mélange est alors abandonné à cette température pendant deux semaines pour favoriser la germination des cristaux. Le résidu final a subi en premier un refroidissement lent (5°/h) jusqu'à 873 K puis un second rapide (50°/h) jusqu'à la température ambiante. Des cristaux jaunâtres, de taille suffisante pour les mesures des intensités, ont été séparés du flux par l'eau bouillante. Une analyse qualitative au M.E.B.E. de type FEI Quanta 200 confirme la présence des différents éléments chimiques attendus: As, Mo, et Na.

### Refinement

L'analyse de la Fourier différence finale ne révèle aucun pic résiduel significatif. Par ailleurs les ellipsoïdes sont très bien définis.

### Crystal data

**Table:**

NaMoO_2_(AsO_4_)	*F*(000) = 536
*M**_r_* = 289.85	*D*_x_ = 3.876 Mg m^−^^3^
Orthorhombic, *P**n**m**a*	Mo *K*α radiation, λ = 0.71073 Å
Hall symbol: -P 2ac 2n	Cell parameters from 25 reflections
*a* = 10.147 (2) Å	θ = 12–15°
*b* = 6.597 (2) Å	µ = 9.29 mm^−^^1^
*c* = 7.420 (2) Å	*T* = 298 K
*V* = 496.7 (2) Å^3^	Prism, yellow
*Z* = 4	0.26 × 0.16 × 0.10 mm

### Data collection

**Table:**

Enraf–Nonius CAD-4 diffractometer	539 reflections with *I* > 2σ(*I*)
Radiation source: fine-focus sealed tube	*R*_int_ = 0.027
graphite	θ_max_ = 27.0°, θ_min_ = 3.4°
ω/2θ scans	*h* = −1→12
Absorption correction: ψ scan (North *et al.*, 1968)	*k* = −1→8
*T*_min_ = 0.186, *T*_max_ = 0.394	*l* = −9→9
1438 measured reflections	2 standard reflections every 120 min
589 independent reflections	intensity decay: 1.2%

### Refinement

**Table:**

Refinement on *F*^2^	Primary atom site location: structure-invariant direct methods
Least-squares matrix: full	Secondary atom site location: difference Fourier map
*R*[*F*^2^ > 2σ(*F*^2^)] = 0.019	*w* = 1/[σ^2^(*F*_o_^2^) + (0.0251*P*)^2^ + 0.2566*P*] where *P* = (*F*_o_^2^ + 2*F*_c_^2^)/3
*wR*(*F*^2^) = 0.053	(Δ/σ)_max_ = 0.001
*S* = 1.14	Δρ_max_ = 0.80 e Å^−^^3^
589 reflections	Δρ_min_ = −0.69 e Å^−^^3^
56 parameters	Extinction correction: *SHELXL97* (Sheldrick, 2008), Fc^*^=kFc[1+0.001xFc^2^λ^3^/sin(2θ)]^-1/4^
0 restraints	Extinction coefficient: 0.0099 (7)

### Special details

**Table:**

Geometry. All e.s.d.'s (except the e.s.d. in the dihedral angle between two l.s. planes) are estimated using the full covariance matrix. The cell e.s.d.'s are taken into account individually in the estimation of e.s.d.'s in distances, angles and torsion angles; correlations between e.s.d.'s in cell parameters are only used when they are defined by crystal symmetry. An approximate (isotropic) treatment of cell e.s.d.'s is used for estimating e.s.d.'s involving l.s. planes.
Refinement. Refinement of *F*^2^ against ALL reflections. The weighted *R*-factor *wR* and goodness of fit *S* are based on *F*^2^, conventional *R*-factors *R* are based on *F*, with *F* set to zero for negative *F*^2^. The threshold expression of *F*^2^ > σ(*F*^2^) is used only for calculating *R*-factors(gt) *etc*. and is not relevant to the choice of reflections for refinement. *R*-factors based on *F*^2^ are statistically about twice as large as those based on *F*, and *R*- factors based on ALL data will be even larger.

### Fractional atomic coordinates and isotropic or equivalent isotropic displacement parameters (Å^2^)

**Table:**

	*x*	*y*	*z*	*U*_iso_*/*U*_eq_	Occ. (<1)
Mo1	0.32396 (4)	0.2500	0.44866 (5)	0.00991 (16)	
As1	0.16109 (5)	0.2500	1.03646 (5)	0.00844 (16)	
Na1	0.5123 (3)	0.0151 (6)	0.8833 (4)	0.0280 (7)	0.50
O1	0.6584 (3)	0.2500	1.0056 (5)	0.0213 (8)	
O2	0.5243 (3)	0.2500	0.3365 (4)	0.0148 (7)	
O3	0.3009 (3)	0.2500	0.1573 (4)	0.0120 (6)	
O4	0.3898 (3)	0.2500	0.6593 (4)	0.0227 (8)	
O5	0.1628 (2)	0.0465 (4)	0.8960 (3)	0.0172 (5)	

### Atomic displacement parameters (Å^2^)

**Table:**

	*U*^11^	*U*^22^	*U*^33^	*U*^12^	*U*^13^	*U*^23^
Mo1	0.0098 (2)	0.0112 (2)	0.0087 (2)	0.000	−0.00007 (14)	0.000
As1	0.0085 (2)	0.0086 (3)	0.0082 (2)	0.000	−0.00054 (16)	0.000
Na1	0.0261 (17)	0.0240 (18)	0.0339 (15)	−0.0048 (12)	−0.0057 (12)	−0.0035 (17)
O1	0.0145 (17)	0.031 (2)	0.0178 (15)	0.000	−0.0009 (13)	0.000
O2	0.0092 (14)	0.0224 (19)	0.0128 (15)	0.000	−0.0022 (13)	0.000
O3	0.0100 (14)	0.0131 (16)	0.0129 (14)	0.000	−0.0022 (12)	0.000
O4	0.0163 (17)	0.039 (2)	0.0124 (14)	0.000	−0.0019 (13)	0.000
O5	0.0231 (13)	0.0128 (11)	0.0158 (10)	−0.0016 (10)	−0.0028 (9)	−0.0042 (10)

### Geometric parameters (Å, °)

**Table:**

Mo1—O4	1.700 (3)	As1—O5	1.700 (2)
Mo1—O1^i^	1.714 (3)	As1—O5^v^	1.700 (2)
Mo1—O5^ii^	1.999 (3)	Na1—O1	2.329 (4)
Mo1—O5^iii^	1.999 (3)	Na1—O2^vi^	2.420 (4)
Mo1—O3	2.174 (3)	Na1—O5^vii^	2.583 (4)
Mo1—O2	2.197 (3)	Na1—O4	2.590 (4)
As1—O2^i^	1.678 (3)	Na1—O1^viii^	2.595 (4)
As1—O3^iv^	1.678 (3)	Na1—O3^vi^	2.597 (4)
			
O4—Mo1—O1^i^	101.71 (16)	O1^i^—Mo1—O2	169.17 (14)
O4—Mo1—O5^ii^	98.82 (7)	O5^ii^—Mo1—O2	82.17 (7)
O1^i^—Mo1—O5^ii^	96.00 (7)	O5^iii^—Mo1—O2	82.17 (7)
O4—Mo1—O5^iii^	98.82 (7)	O3—Mo1—O2	73.93 (12)
O1^i^—Mo1—O5^iii^	96.00 (7)	O2^i^—As1—O3^iv^	113.51 (16)
O5^ii^—Mo1—O5^iii^	156.15 (12)	O2^i^—As1—O5	110.67 (10)
O4—Mo1—O3	163.05 (14)	O3^iv^—As1—O5	108.60 (10)
O1^i^—Mo1—O3	95.24 (14)	O2^i^—As1—O5^v^	110.67 (10)
O5^ii^—Mo1—O3	79.22 (6)	O3^iv^—As1—O5^v^	108.60 (10)
O5^iii^—Mo1—O3	79.22 (6)	O5—As1—O5^v^	104.37 (16)
O4—Mo1—O2	89.12 (14)		

Symmetry codes: (i) *x*−1/2, *y*, −*z*+3/2; (ii) −*x*+1/2, −*y*, *z*−1/2; (iii) −*x*+1/2, *y*+1/2, *z*−1/2; (iv) *x*, *y*, *z*+1; (v) *x*, −*y*+1/2, *z*; (vi) −*x*+1, −*y*, −*z*+1; (vii) *x*+1/2, *y*, −*z*+3/2; (viii) −*x*+1, −*y*, −*z*+2.
